# Comparison of raw and processed *Radix Polygoni Multiflori *(*Heshouwu*) by high performance liquid chromatography and mass spectrometry

**DOI:** 10.1186/1749-8546-5-29

**Published:** 2010-08-12

**Authors:** Zhitao Liang, Hubiao Chen, Zhiling Yu, Zhongzhen Zhao

**Affiliations:** 1 School of Chinese Medicine, Hong Kong Baptist University, Kowloon, Hong Kong SAR, China

## Abstract

**Background:**

*Radix Polygoni Multiflori *is the dried root tuber of *Polygonum multiflorum *Thunb. (Fam. Polygonaceae). According to Chinese medicine theory, raw (R-RPM) and processed (P-RPM) *Radix Polygoni Multiflori *possess different properties. The present study investigates the differences in chemistry between raw and processed *Radix Polygoni Multiflori*.

**Methods:**

Five pairs of R-RPM and P-RPM as well as 15 commercial decoction pieces were analyzed with high performance liquid chromatography (HPLC) and mass spectrometry (MS).

**Results:**

Two anthraquinones, namely emodin-8-*O*-(6'-*O*-malonyl)-glucoside and physcion-8-*O*-(6'-*O*-malonyl)-glucoside disappeared or decreased significantly and 2,3,5,4'-tetrahydroxystilbene-2-*O*-*β*-*D*-glucopyranoside, emodin-8-*O*-*β*-*D*-glucopyranoside and physcion-8-*O*-*β*-*D*-glucopyranoside decreased after the R-RPM samples being processed. On the other hand, the contents of emodin and physcion generally increased after processing.

**Conclusion:**

The present study indicates that processing *Radix Polygoni Multiflori *may change the contents and types of chemicals in it. These changes are probably responsible for the various pharmacological effects of R-RPM and P-RPM as well as hepatotoxicity.

## Background

Proper pharmaceutical processing may reduce toxicity or side effects, potentiate the beneficial effects, change the pharmacological properties, preserve active constituents, facilitate administration, improve flavor or correct unpleasant taste and increase purity of Chinese *materia medica *[1-4]. In China, the processing methods for *Radix Polygoni Multiflori *have been practiced since the Tang dynasty [5] and are documented in the Chinese pharmacopoeia [6]. *Radix Polygoni Multiflori *(*Heshouwu*) is the dried root tuber of *Polygonum multiflorum *Thunb. (Fam. Polygonaceae) [6]. According to Chinese medicine theory, raw *Radix Polygoni Multiflori *(R-RPM) counteracts toxicity, cures carbuncles and relaxes the bowels whereas processed *Radix Polygoni Multiflori *(P-RPM) replenishes the liver and kidney with vital essence and blood, blackens the hair and strengthens the tendons and bones.

R-RPM and P-RPM possess different pharmacological properties. While P-RPM (steamed with black bean juice) enhanced immune activities and anti-immunosuppression, R-RPM did not [7]. R-RPM was purgative whereas P-RPM was not [8], probably due to lower content of anthraquinones glycosides in P-RPM. R-RPM inhibited triglyceride accumulation induced by carbon tetrachloride (CCl_4_), cortisone acetate and thioacetamide (TAA) in the mouse liver and P-RPM lowered the triglyceride accumulation induced by cortisone acetate; both R-RPM and P-RPM reduced liver enlargement caused by CCl_4 _[9].

It is important to differentiate R-RPM from P-RPM because *Radix Polygoni Multiflori *was linked to hepatotoxicity and other liver conditions [10-15]. Over-the-counter preparations such as *Shouwu pian *and *Shenmin *(both containing *Radix Polygoni Multiflori*) may cause acute hepatitis. A recent study found that, *Radix Polygoni Multiflori *was the hepatotoxic component that caused acute hepatitis [16]. There were other hepatotoxic cases related to *Radix Polygoni Multiflori *[17-20]. R-RPM did not induce liver injury [21] but P-RPM could damage rat's liver after long-term use of high dosages (40 g/kg/day) by intragastric administration. However, no toxic or side effects were found when P-RPM was used at the dosage of 22 g/kg/day which is 10 times of the normal intake for adult per day [22,23].

*Radix Polygoni Multiflori *contains anthraquinones (emodin, chrysophanol, physcion, citreorosein, chrysophanol-8-*O*-*β*-*D*- glucopyranoside, physcion-8-*O*-*β*-*D*-glucopyranoside, emodin-8-*O*-*β*-*D*- glucopyranoside, emodin-1,6-dimethylether, questin, questinol, 2-acetylemodin, 2-methoxy-6-acetyl-7-methyljuglone, emodin-8-*O*-(6'-*O*-malonyl)-glucoside) [24-26]; stilbene glucosides (2,3,5,4'-tetrahydroxystilbene-2-*O*-*β*-*D*-glucopyranoside, 2,3,5,4'- tetrahydroxystilbene-2, 3-*O*-*β*-*D*- glucopyranoside [27]) and flavonoids (tricin [25], quercetin-3-*O*-galactoside, quercetin-3-*O*-arabinoside [28]), as well as gallic acid, catechin [29], torachrysone-8-*O*-*β*-*D*-glucopyranoside [27], N-transferuloyl tyramine, N-transferuloyl-3-methyldopamine [25] and 1,3-dihydroxy-6,7 -dimethylxanthone -1-*O*-*β*-*D*-glucopyranoside [27]. There were more free anthraquinones in P-RPM than that in R-RPM. However, anthraquinone glycosides and stilbene glucoside were more abundant in R-RPM than P-RPM [30]. P-RPM contains components not present in R-RPM, namely 2,3-dihydro-3,5-dihydroxy-6-methyl-4(H)-pyran-4-one and 5-hydroxymethyl furfural; P-RPM contains less amino acids and monosaccharides and has a lower pH value than R-RPM [31].

In recent years, high performance liquid chromatography (HPLC) and gas chromatography (GC) have been employed to determine the level of anthraquinones in *Radix Polygoni Multiflori *[32,33].

Using HPLC-DAD and mass spectrometry, the present study compares five pairs of raw and processed *Radix Polygoni Multiflori *as well as some samples from commercially available decoctions.

## Methods

### Plants

Five samples of R-RPM and 15 samples of commercial decoction pieces of *Radix Polygoni Multiflori *were collected from cultivation areas or purchased from pharmacies in China (Table 1). The R-RPM was softened by water and then steamed in an autoclave (HV-85, Hirayama, Japan) for four hours at 121☐ and under 2.03 pounds per square inch (psi), according to the processing methods documented in the Chinese pharmacopoeia [6]. All the herbs were authenticated macroscopically by Prof Zhongzhen Zhao. The corresponding voucher specimens were deposited in the Bank of China (Hong Kong) Chinese Medicines Centre of Hong Kong Baptist University, Hong Kong SAR, China.

**Table 1:** A list of tested samples from China

Sample name	No.	Source	Collection time
Raw *Radix Polygoni Multiflori*	1	Daqiao Village, Deqing County, Guangdong, China; cultivated	2008. 05. 30
	2	Dengyun Village, Deqing County, Guangdong, China; cultivated	2008. 05. 30
	3	Duimian Village, Deqing County, Guangdong, China; cultivated	2008. 05. 30
	4	Chengdu, Sichuan, China; market	2008. 09. 25
	5	Guangzhou, Guangdong, China; market	2008. 12. 10
Commercial *Radix Polygoni Multiflori *from Deqing County, Guangdong, China	1	Wild	2007. 12. 25
	2	Half wild for 5-6 years	2007. 12. 25
	3	Cultivated in the mountain for 5-6 years	2007. 12. 25
	4	Cultivated in the normal soil for 3-4 years	2007. 12. 25
	5	Cultivated in the mountain	2007. 12. 25
	6	Cultivated in the normal soil for one year	2007. 12. 25
	7	Cultivated in the normal soil for one year	2007. 12. 25
	8	Cultivated in the normal soil for one year	2007. 12. 25
Commercial processed *Radix Polygoni Multiflori *from Chinese herbal shops	1	Hong Kong, China; market	2007. 12. 05
	2	Hong Kong, China; market	2007. 12. 05
	3	Hong Kong, China; market	2007. 12. 05
	4	Hong Kong, China; market	2007. 12. 05
	5	Shenzhen, Guangdong, China; market	2007. 12. 05
	6	Shenzhen, Guangdong, China; market	2007. 12. 05
	7	Guangzhou, Guangdong, China; market	2008. 12. 10

### Instrumentation

A CREST 1875HTAG ultrasonic processor (CREST, USA) was used for sample extraction. HPLC fingerprinting analysis was performed on an Agilent1100 series LC system consisting of a G1311A Quart pump, a G1322A degasser, a G1315A photodiode array detector (DAD) and a G1313A automatic liquid sampler (ALS). A MicroQTOF system with an electrospray ionization source (Bruker Daltonics, Germany) was used for mass spectrometric analysis. Separation was performed at room temperature on an Alltima C_18 _analytical column (250 mm × 4.6 mm, 5 μm, Alltech Associates, USA) coupled with a C_18 _guard column (7.5 mm × 4.6 mm, 5 μm, Alltech Associates, USA) that was eluted with acetonitrile (containing 0.5% acetic acid)/water (containing 0.5% acetic acid) at a flow rate of 1 mL/min by a discontinuous gradient in which acetonitrile was adjusted to 10%, 35% and 100%, at 0, 45 and 65 minutes respectively. Detection was performed at 280 nm. The mass spectra were detected in positive mode. The flow rate of drying gas (N_2_) and nebulizing gas were 4 L/min and 0.4 L/min respectively. Ion source temperature was set at 200☐ and the scan range was 200-1500 amu.

### Chemicals and reagents

HPLC-grade acetonitrile (Labscan, Thailand) and deionized water obtained from a Milli-Q water system (Millipore, USA) were used for preparation of the mobile phase. Analytical grade methanol (Labscan, Thailand) was used for preparation of standards and sample extraction. Reference compounds of 2,3,5,4'-tetrahydroxystilbene-2-O-*β*-*D*- glucopyranoside (THSG, **1**), emodin (**2**) and physcion (**3**) (purities >97%) were purchased from the National Institute for the Control of Pharmaceutical and Biological Products, China (Batch numbers 110844-200505, 110756-200110 and 110758-200610 respectively).

### Preparation of standard and sample solutions

The three reference compounds (**1**-**3**) were accurately weighed and dissolved in methanol to produce standard solutions. 0.5 g powdered sample was refluxed with 25 ml methanol for 90 minutes. Then the supernatant was filtered through a 0.45 μm membrane and 10 μl samples were analyzed with HPLC and LC-MS.

### Method validation

Reproducibility and repeatability of the method were determined with five injections of one sample solution and five replicates of one solid sample prepared according to the method. Stability of the method was determined with the sample solution after 0, 2, 4, 8 and 12 hours in a single day and for further one and two days.

### Data processing

Chromatographic data were analyzed with Computer Aided Similarity Evaluation System software (Central South University, China) [34]. The software synchronized the chromatographic peaks and calculated the correlation coefficients for similarity of the chromatograms.

## Results and discussion

### Optimization and validation of HPLC conditions

To optimize the elution conditions, we investigated the mobile phase of acetonitrile (containing 0.5% acetic acid)-water (containing 0.5% acetic acid) with various gradients and the optimal acetonitrile-water system was determined to have acetonitrile adjusted to 10%, 35%, and 100%, at 0, 45 and 65 min, respectively.

The limits of detection, evaluated by a signal-to-noise ratio of about 3:1 for the standard solution, were 0.575 μg/ml, 0.343 μg/ml and 0.523 μg/ml for compounds **1**, **2 **and **3 **respectively. The correlation coefficients were 0.973 ± 0.021 (*n *= 5) at 280 nm detection wavelength for reproducibility and 0.968 ± 0.022 (*n *= 5) for repeatability test. In stability testing, the correlation coefficients were 0.972 ± 0.034 (*n *= 5) over a period of 12 hours and 0.984 ± 0.015 (*n *= 7) over a period of three days. These results indicated that the conditions for the fingerprint analysis were satisfactory.

### Comparison of R-RPM and P-RPM fingerprints

**Figure 1:**
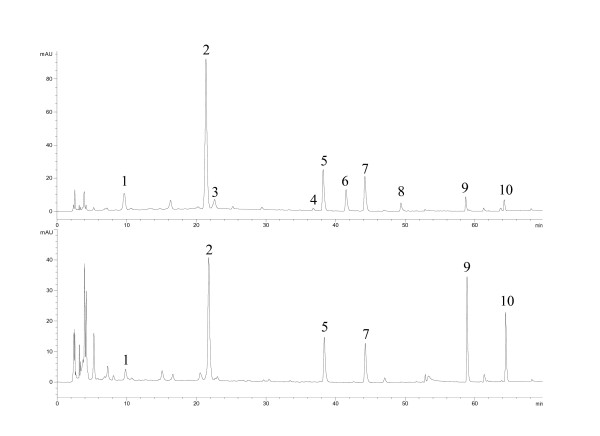
**HPLC chromatograms of raw and processed *Radix Polygoni Multiflori *from Dengyun Village, Deqing County, Guangdong, China (refer to Table 2 for peak numbering)**.

**Figure 2:**
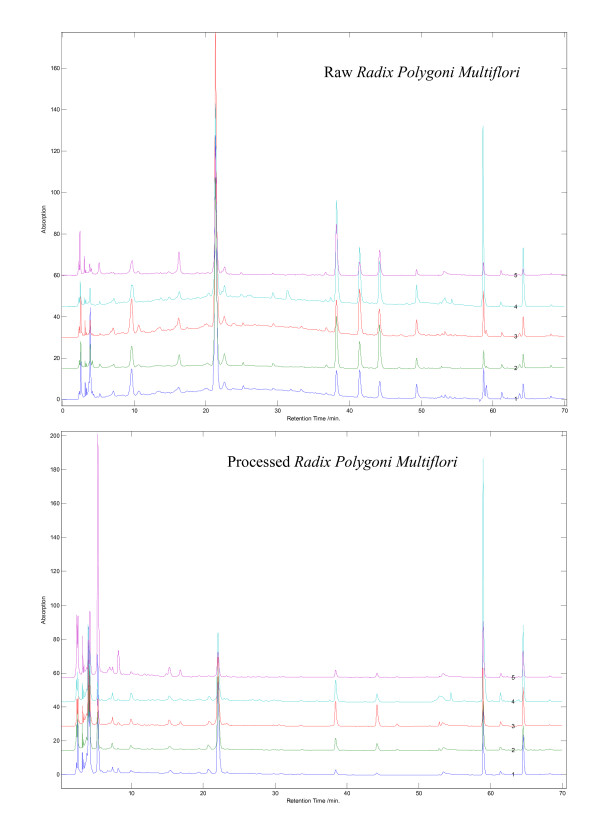
**HPLC fingerprints of R-RPM and its corresponding P-RPM from various sources in China**.

**Figure 3:**
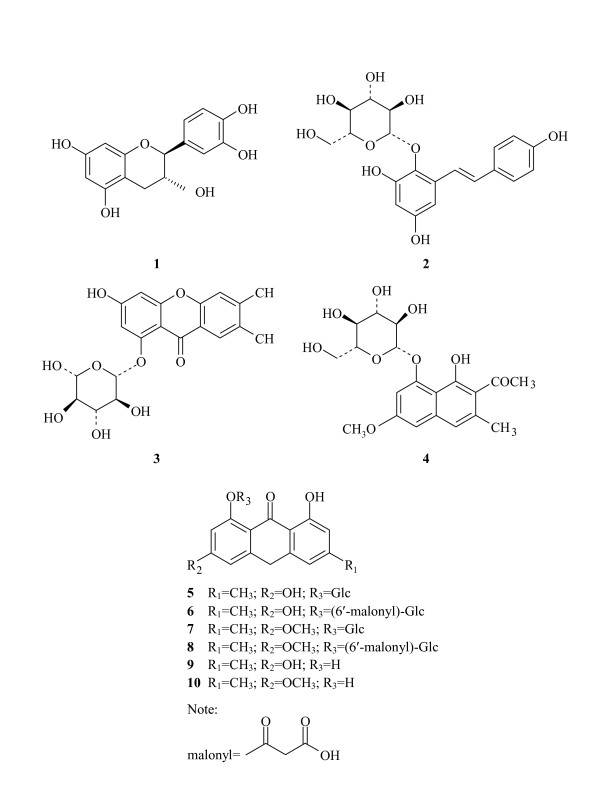
**Chemical structures of the identified compounds in the HPLC chromatograms Peak 1: catechin; Peak 2: 2,3,5,4'-tetrahydroxystilbene-2-O-*β*-D- glucopyranoside; Peak 3: 1,3-dihydroxy-6,7-dimethylxanthone-1-*O*-*β*-*D*-glucopyranoside; Peak 4: torachrysone-8-*O*-*β*-*D*-glucopyranoside; Peak 5: emodin-8-*O*-*β*-*D*- glucopyranoside; Peak 6: emodin-8-(6'-*O*-malonyl)-glucoside; Peak 7: physcion-8-*O*-*β*-*D*- glucopyranoside; Peak 8: physcion-8-*O*-(6'-*O*- malonyl)-glucoside; Peak 9: emodin; Peak 10: physcion**.

Five samples of R-RPM and their corresponding P-RPM were analyzed. Chromatograms for R-RPM and P-RPM were visually distinguishable from each other (Figures 1 and 2). In the chromatograms of R-RPM, there were ten well-separated chromatographic peaks (Figure 1). Chromatographic peaks 2, 9 and 10 were unambiguously identified as 2,3,5,4'-tetrahydroxystilbene-2-*O*-*β*-*D*-glucopyranoside (THSG), emodin and physcion respectively. Chromatographic peaks 1, 3, 4, 5, 6, 7 and 8 were tentatively identified as catechin, 1,3-dihydroxy-6,7-dimethylxanthone- 1-*O*-*β*-*D*-glucopyranoside, torachrysone-8-*O*-*β*-*D*-glucopyranoside, emodin-8-*O*-*β*-*D*- glucopyranoside, emodin-8-(6'-*O*-malonyl)-glucoside, physcion-8-*O*-*β*-*D*- glucopyranoside and physcion-8-*O*-(6'-*O*- malonyl)-glucoside [26,27,29]. The exact *cis*/*trans *configuration of catechin was not identified. Moreover, physcion-8-*O*-(6'-*O*-malonyl)-glucoside was identified in R-RPM for the first time (Table 2 and Figure 3).

**Table 2:** MS data of major identified/unknown compounds in the HPLC chromatograms of R-RPM

Peak No.	Mass Spectra	Identified compounds (tentative names)
1	291.1 ([M+H]^+^); 581.2 ([2M+H]^+^)	Catechin
2	407.1 ([M+H]^+^)	2,3,5,4'-tetrahydroxystilbene-2-*O*-*β*-*D*- glucopyranoside
3	257.1 ([M+H-glu]^+^); 419.1 ([M+H]^+^)	1,3-dihydroxy-6,7-dimethylxanthone-1- *O*-*β*-*D*- glucopyranoside
4	247.1 ([M+H-glu]^+^); 409.1 ([M+H]^+^); 431.1 ([M+Na]^+^)	Torachrysone-8- *O*-*β*-*D*- glucopyranoside
5	271.1 ([M+H-glu]^+^); 455.1 ([M+Na]^+^)	Emodin-8-*O*-*β*-*D*-glucopyranoside
6	271.1 ([M+H-malonyl-glu]^+^); 541.1 ([M+Na]^+^); 1059.2 ([2M+K]^+^)	Emodin-8-(6'-*O*-malonyl)-glucoside
7	285.1 ([M+H-glu]^+^); 469.1 ([M+Na]^+^)	Physcion-8-*O*-*β*-*D*- glucopyranoside
8	285.1 ([M+H-malonyl-glu]^+^); 555.1 ([M+Na]^+^); 1103.2 ([2M+K]^+^)	Physcion-8-*O*-(6'-*O*-malonyl)-glucoside
9	271.1 ([M+H]^+^)	Emodin
10	285.1 ([M+H]^+^)	Physcion

**Figure 4:**
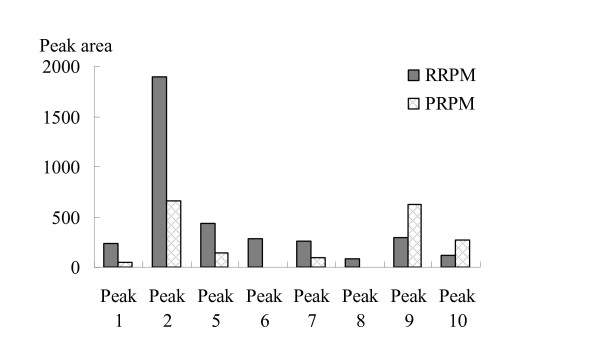
**The change of relative contents of main compounds between R-RPM and their corresponding P-RPM**.

The chromatograms of R-RPM showed that catechin, THSG and anthraquinones glycosides were the main components. The concentrations of these constituents decreased greatly after being processed. Emodin-8-*O*-(6'-*O*-malonyl)-glucoside and physcion-8-*O*-(6'-*O*-malonyl)-glucoside disappeared or decreased greatly in the processed products (Figures 1 and 2). Meanwhile, catechin, THSG, emodin-8-*O*-*β*-*D*-glucopyranoside and physcion-8-*O*-*β*-*D*-glucopyranoside decreased among five of the tested samples (Figure 4). On the other hand, the contents of emodin and physcion increased on average. The change of emodin-8-*O*-(6'-*O*-malonyl)-glucoside, physcion-8-*O*-(6'-*O*-malonyl)-glucoside, emodin-8-*O*-*β*-*D*-glucopyranoside and physcion-8-*O*-*β*-*D*-glucopyranoside probably contributed to the increase of emodin and physcion. The results indicated that heating made anthraquinones glycosides lose their glycosides and that the ratio of free anthraquinones to anthraquinones glycosides increased greatly while the ratio of THSG to free anthraquinones decreased. The change in type, amount and ratio of chemical components is probably responsible for the different functions and pharmacological effects of R-RPM and P-RPM.

### Comparison of fingerprints of commercial Radix Polygoni Multiflori

**Figure 5:**
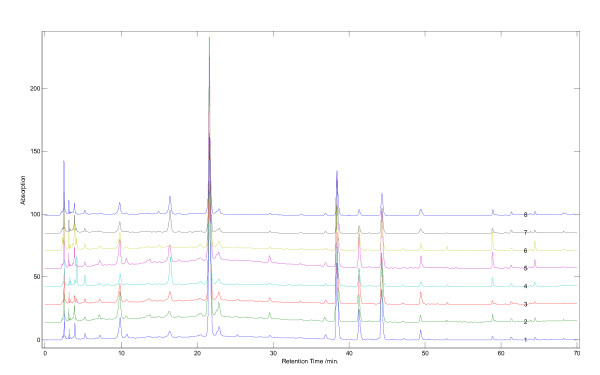
**HPLC fingerprints of commercial decoction pieces of *Radix Polygoni Multiflori *from Deqing County, Guangdong, China**.

**Figure 6:**
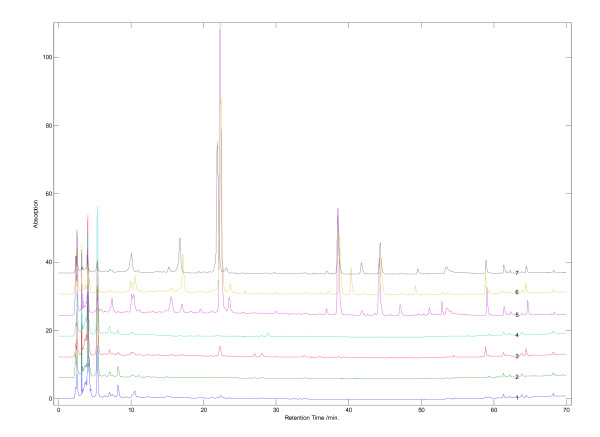
**HPLC fingerprints of commercial P-RPM purchased from Chinese herb shops in Hong Kong, Shenzhen and Guangzhou**.

In Deqing County, Guangdong, China (considered genuine production area for *Radix Polygoni Multiflori*), we purchased several grades of commercial decoction pieces of *Radix Polygoni Multiflori *at the local herb markets (Table 1). The correlation coefficients for the fingerprints were 0.978 ± 0.012 (*n *= 8), suggesting that the samples were very similar among them (Figure 5). We further compared seven batches of samples purchased from pharmacies in Hong Kong, Shenzhen and Guangzhou. Unfortunately, the correlation coefficients were 0.671 ± 0.116 (*n *= 8), suggesting that the samples varied significantly in both content and chemicals among these P-RPM samples (Figure 6). For example, the samples from Hong Kong were over-processed, drastically reducing the content of THSG, emodin-8-*O*-(6'-*O*-malonyl)-glucoside and physcion-8-*O*-(6'-*O*-malonyl)-glucoside which were present in all the samples from Shenzhen and Guangzhou.

## Conclusion

The present study demonstrates that processing *Radix Polygoni Multiflori *may change the contents, particularly the quantity and types of chemicals in it. These changes are probably responsible for the various pharmacological effects of R-RPM and P-RPM as well as hepatotoxicity.

We report here for the first time the disappearance or significant decrease of the two glucosides, emodin-8-*O*-(6'-*O*-malonyl)-glucoside and physcion-8-*O*-(6'-*O*- malonyl)-glucoside, during the processing of R-RPM. These two compounds may be used as chemical markers for differentiating R-RPM from P-RPM. In addition, these two compounds together with emodin-8-*O*-*β*-*D*-glucopyranoside, physcion-8-*O*-*β*-*D*-glucopyranoside, emodin and physcion may be used as chemical markers for the quality control of R-RPM; the latter four compounds may be used to assess the quality of P-RPM.

## Abbreviations

R-RPM: raw *Radix Polygoni Multiflori*; P-RPM: processed *Radix Polygoni Multiflori*; HPLC: high performance liquid chromatography; MS: mass spectrometry; THSG: 2,3,5,4'-tetrahydroxystilbene-2-*O*-*β*-*D*-glucopyranoside

## Competing interests

The authors declare that they have no competing interests.

## Authors' contributions

ZY and HC designed the study. ZL conducted the experiments and drafted the manuscript. ZZ supervised the study and revised the manuscript. All authors read and approved the final version of the manuscript.
